# Impact of self-perceived oral health and socio-economic factors on oral health-related behavior in Estonian adults

**DOI:** 10.2340/aos.v83.41902

**Published:** 2024-09-25

**Authors:** Marjo Sinijärv, Jana Olak, Rein Murakas, Riina Runnel

**Affiliations:** a Institute of Dentistry, Faculty of Medicine, University of Tartu, Tartu, Estonia; b Stomatology Clinic, Tartu University Hospital, Tartu, Estonia; c Faculty of Arts and Humanities, University of Tartu, Tartu, Estonia; d Rein Murakas Consulting, Tartu, Estonia

**Keywords:** Oral hygiene, oral health, health-related behavior, population

## Abstract

**Objective:**

To identify which socioeconomic factors are affecting oral health-related behavior and to provide suggestions for improving the population’s oral health.

**Materials and methods:**

The survey includes population groups from age 35 and older from all 15 Estonian counties and major cities (*n* = 2,376). The study is based on data from a nationwide Estonian Adult Oral Health Survey questionnaire. In addition to analyzing eight aspects of oral health-related behavior and self-perceived oral health variables, the survey also includes participants’ socio-economic and demographic characteristics. The study utilizes frequency tables (including cumulative distributions), means, correlations, and regression analysis as its methods.

**Results:**

The mean number of beneficial behaviors reported by the participant was 4.2 (SD 1.6). The value of the oral health-related behavior index (OHBI, the number of reported behaviors from the eight) is initially determined by the optimal timing between meals, abstinence from smoking, and the choice of drinking water or refraining from any intake between meals. Participants with higher OHBI tended to rate their self-perceived oral health better. Adherence to beneficial dental health-related behavior in Estonian adults is primarily influenced by gender, educational level, type of settlement, and household income level.

**Conclusions:**

In order to significantly improve oral health and related behaviors, it is imperative to integrate dental services into universal health coverage and deliver ongoing oral health education for adults.

## Introduction

Oral health is a significant global public health concern, impacting billions of individuals across the globe [1] and affecting half of the European population [2]. The burden of oral diseases is unevenly distributed, particularly impacting socio-economically disadvantaged and marginalized societal groups. This inequality is closely linked with socio-economic status and broader social determinants of health [3]. Although systemic health conditions can impact oral health outcomes [4], the key factors for achieving optimal oral health are primarily linked to an individual’s commitment to personal dental care practices and the decreasing (or removal) of barriers to accessing dental care [5].

While the consideration of older age plays a significant role in shaping public dental coverage policies in many countries, some nations do not consider advanced age as a strict eligibility criterion for such coverage. Instead, they provide different levels of coverage based on meeting specific low-income thresholds [6] or other country specific models [7].

The National Institute for Health Development conducts a biennial population-based survey Health Behavior among Estonian Adult Population aged 16–64. According to the survey, 66.7% of respondents (55.0% of men and 75.0% of women) reported brushing their teeth twice a day (or more often), while 5.5% reported brushing their teeth less than once a day. The survey also found that lower levels of education and income are associated with poorer oral hygiene [8]. Individuals who report infrequent tooth brushing demonstrate a higher risk for the onset or progression of dental caries compared to those with more regular brushing habits; the impact was more noticeable in permanent teeth [9]. Additional variables, such as diet [10], frequency of dental visits [11], knowledge of oral health and hygiene [12], and smoking [13], also affect oral health.

The study aimed to identify the socio-economic factors affecting oral health-related behavior of Estonian adults and provide suggestions to policymakers for improving the population’s oral health.

## Materials and methods

### Study design

The study utilized data from a nationwide Estonian Adult Oral Health Survey questionnaire. Information on adults’ oral health was collected through a self-administered questionnaire filled in electronically by the participants. Data collection took place from April 2019 to September 2021. The methodology of the present study was partially adapted from the previous European studies that assessed oral health-related behaviors [14, 15].

### Study population

The survey aimed to include all population groups aged 35 or older from all 15 Estonian counties and major cities. Participants were recruited through public channels by the survey’s partner institutions. Information about the ongoing survey and invitations to participate were shared in the press, social media, and with local municipalities, healthcare providers, employers, medical and patient associations, and social welfare institutions. The survey sample aimed to represent the adult population of Estonia aged 35 and older based on gender, age, and territorial distribution across Estonian counties. Due to the COVID-19 pandemic, the territorial distribution could not be fully realized; however, all Estonian counties and major cities were represented in the sample.

The survey participants completed the questionnaire electronically in either Estonian or Russian. Paper questionnaires were also available, data from which the researcher later entered into the database.

The survey database comprised 2,709 electronic questionnaires, of which 2,376 were included in this study as they included all background characteristics (gender, age group, language of response, educational level, settlement type, income group) without data gaps (missing values). A total of 2,247 questionnaires had no missing data regarding oral health-related behavior components included in this study.

Based on the data, the participants were divided into five age- and gender-based (male and female) sub-groups (35–44M, 35–44F, 45–54M, 45–54F, 55–64M, 55–64F, 65–74M, 65–74F, 75+M, 75+F).

### Self-reported socio-economic and demographic characteristics

Data on educational level, type of settlement, language of instruction, and subjective assessment of household income level were included as socio-economic and demographic characteristics. Educational level was categorized into primary or less, secondary (including vocational secondary education), and tertiary/higher (including applied higher education). The type of settlement as a possible determinant of access to oral health services was included as Tallinn (capital city), larger Estonian cities (Tartu, Pärnu, Kohtla-Järve, Narva), other smaller cities/towns, small settlements (villages), or countryside. The language of instruction was defined from the submitted questionnaire (in Estonian or Russian). The subjective assessment of household’s income level was divided into four self-assessed categories: *living comfortably on present income*, *coping on present income*, *difficult on present income*, and *very difficult on present income*. The answer *cannot say* was treated as a missing value. This indicator was previously used in the European Social Survey (ESS) and in various Estonian adult population surveys.

### Self-reported oral health-related behavior

The study included data on eight aspects of oral health-related behavior: the intervals between meals, main drink between meals, toothbrushing frequency, frequency of skipping routine toothbrushing for various reasons, interdental cleaning, getting recommendations/education on oral health, dental check-up intervals, and smoking habits. The most favorable oral health-related behavior includes the following self-assessed habits: the interval between meals of 3 h or more; consumption of tap water as a main drink between meals; toothbrushing frequency twice daily; never missing daily toothbrushing; daily interdental cleaning; received advice on oral hygiene or diet in connection with dental health; visited the dentist for a check-up in the last 12 months; and non-smoking (see Table 2). The count (from 0 to 8) of beneficial behaviors followed by the participant was described as an oral health-related behavior index (OHBI).

### Self-perceived oral health

Self-perceived oral health included the following variables from the survey: self-perceived dental/oral health reported as very good; no new cavities formed during last year; dental and oral problems have not caused changes in diet; possible to eat nuts/apple/cucumber/raw carrot without cutting them into pieces; absence of pain or discomfort in the last couple of years related to teeth or surrounding tissues; absence of bleeding gums, no teeth extracted; based on self-assessment no dental treatment is required.

### Ethics statement

The survey was conducted in accordance with the World Medical Association Declaration of Helsinki [16] and followed World Health Organization guidelines [17], and was approved by the University of Tartu Research Ethics Committee (No 291/T-6; Annex 293/M-9; 301/M-9) and Tartu University Hospital Clinical Ethics Committee (No 19055).

Participation in the survey was voluntary, and participants signed a written informed consent form before the study.

### Statistical analysis

Statistical data analysis was performed using IBM SPSS Statistics. In the variables overview, one- and multi-dimensional frequency tables (including cumulative distributions) were utilized and means compared. A correlation analysis was conducted to examine the relationship between OHBI and self-perceived oral health indicators. Additionally, regression analysis was used to assess the impact of socio-demographic predictors on the OHBI value. Graphical output was created using MS Excel.

## Results

### General characteristics

**Figure 1:**
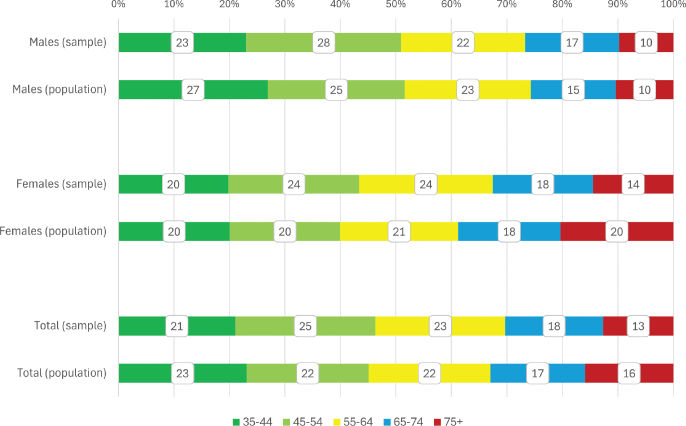
Distribution of study participants (%) by age and gender compared to the overall population size. The population description is based on the population data of Statistics Estonia as of January 1, 2019. Sample (*n* = 2,376): M = 920 (39%), F = 1,456 (61%), population (*N* = 793 771): M = 351 078 (44%), F = 442,693 (56%).

The gender and age-based distribution of the sample generally corresponds to the Estonian adult population (Figure 1).

The distribution of the participants included in the study according to their gender, socio-economic, and demographic characteristics is summarized in Table 1. The majority of participants had secondary education and resided in smaller towns or the countryside. Most participants completed the questionnaire in Estonian language. Over 60% of the participants reported that they were coping with their current income. According to Chi Square test, all surveyed characteristics except for the type of settlement were gender-related (*p* < 0.001).

**Table 1:** Distribution of the participants (*n* = 2,376) by gender and socio-economic and demographic characteristics.

Characteristic	Category	Gender		Total
		Male	Female	
Age group	35–44	212	289	501
		23%	20%	21%
	45–54	257	343	600
		28%	24%	25%
	55–64	205	350	555
		22%	24%	23%
	65–74	156	263	419
		17%	18%	18%
	75+	90	211	301
		10%	15%	13%
	Total	920	1,456	2,376
		100%	100%	100%
Pearson Chi-Square 18.01, df 4, asymptotic significance (2-sided) 0.001				
Educational level	Primary or less	168	143	311
		18%	10%	13%
	Secondary	547	841	1,388
		60%	58%	58%
	Tertiary	205	472	677
		22%	32%	29%
	Total	920	1,456	2,376
		100%	100%	100%
Pearson Chi-Square 51.28, df 2, asymptotic significance (2-sided) < 0.001				
Type of settlement	Tallinn (capital city)	117	224	341
		17%	17%	17%
	Larger cities (Tartu, Pärnu, Kohtla-Järve, Narva)	158	242	400
		17%	17%	17%
	Other cities, towns	322	522	844
		35%	36%	36%
	Small settlements (villages), countryside	323	468	791
		35%	32%	33%
	Total	920	1,456	2,376
		100%	100%	100%
Pearson Chi-Square 4.50, df 3, asymptotic significance (2-sided) 0.212				
Language of instruction	Estonian	784	1,155	1,939
		85%	79%	82%
	Russian	136	301	437
		15%	21%	18%
	Total	920	1,456	2,376
		100%	100%	100%
Pearson Chi-Square 13.03, df 1, asymptotic significance (2-sided) < 0.001				
Household’s income level (subjective assessment ‘Feeling about household’s income nowadays’)	Living comfortably on current income	154	208	362
		17%	14%	15%
	Coping on current income	599	903	1,502
		65%	62%	63%
	Difficult on current income	125	239	364
		14%	16%	15%
	Very difficult on current income	42	106	148
		5%	7%	6%
	Total	920	1,456	2,376
		100%	100%	100%
Pearson Chi-Square 12.69, df 3, asymptotic significance	0.005			

**Table 2:** Distribution of the less and more beneficial oral health-related behaviors reported by gender (*n* = 2,247). Response options reflected in the index value are indicated in bold.

Characteristic	Category	Gender		Total
		Male	Female	
Interval between meals	**Three hours or more**	738	1,130	1,868
		86%	81%	83%
	Less than 3 h	120	259	379
		14%	19%	17%
Pearson Chi-Square 8.22, df 1, asymptotic significance 0.004				
Main drink between meals	**Water or nothing**	416	928	1,344
		49%	67%	60%
	Other	442	461	903
		52%	33%	40%
Pearson Chi-Square 74.10, df 1, asymptotic significance < 0.001				
Toothbrushing frequency	**Twice daily**	411	953	1,364
		48%	69%	61%
	Less than twice daily	447	436	883
		52%	31%	39%
Pearson Chi-Square 95.35, df 1, asymptotic significance < 0.001				
Missing daily toothbrushing when tired, etc.	**Never**	301	652	953
		35%	47%	42%
	Other answer	557	737	1,294
		65%	53%	58%
Pearson Chi-Square 30.54, df 1, asymptotic significance < 0.001				
Interdental cleaning frequency	**Daily**	125	311	436
		15%	22%	19%
	Less often	733	1,078	1,811
		85%	78%	81%
Pearson Chi-Square 20.75, df 1, asymptotic significance < 0.001				
Received recommendations on oral hygiene and diet	**Yes**	309	606	915
		36%	44%	41%
	No	549	783	1,332
		64%	56%	59%
Pearson Chi-Square12.74, df 1, asymptotic significance < 0.001				
Visited a dentist for a dental and oral check-up (not for a specific ailment)	**In last 12 months**	211	476	687
		25%	34%	31%
	Earlier or not at all	647	913	1,560
		75%	66%	69%
Pearson Chi-Square 23.40, df 1, asymptotic significance < 0.001				
Smoking	**Non-smoking**	632	1,173	1,805
		74%	84%	80%
	Other answer	226	216	442
		26%	16%	20%
Pearson Chi-Square 39.08, df 1, asymptotic significance	< 0.001			

The gender-based distribution of oral health-related behaviors indicates that female respondents showed a more beneficial oral health-related behavior than males, except for more frequent between-meal snacking (Table 2).

The lower OHBI values (0–3) were initially determined by the optimal timing between meals, abstinence from smoking, and the choice of drinking water or abstaining from any intake between meals. The participants who had reported three beneficial oral health-related behaviors among the eight possible, the one most frequently reported (33%) was optimal timing between meals, followed by abstinence from smoking (29%) and drinking water or nothing between meals (24%). Brushing teeth twice daily became an essential variable for participants who reported 4 beneficial oral health-related behaviors. Higher OHBI values (5 or more) were obtained by participants who had received advice on oral hygiene and diet. The final components to be incorporated are interdental cleaning and regular dental check-ups (Table 3).

**Table 3:** Cumulative distribution of the beneficial oral health-related behaviors reported by the participants (%, *n* = 2,247).

Characteristic	Category counted in OHBI	OHBI value							
		1	2	3	4	5	6	7	8
Interval between meals	Three hours or more	3%	13%	33%	55%	76%	91%	98%	100%
Smoking	Non-smoking	1%	9%	29%	52%	74%	90%	99%	100%
Main drink between meals	Water or nothing	0%	6%	24%	46%	68%	87%	98%	100%
Toothbrushing frequency	Twice daily	0%	4%	16%	39%	66%	86%	98%	100%
Missing daily toothbrushing when tired, etc.	Never	0%	2%	11%	30%	57%	82%	97%	100%
Received recommendations on oral hygiene and diet	Yes	0%	4%	14%	34%	58%	83%	97%	100%
Visited a dentist for a dental and oral check-up (not for a specific ailment)	In last 12 months	0%	2%	10%	26%	50%	78%	96%	100%
Interdental cleaning frequency	Daily	0%	2%	9%	20%	44%	70%	93%	100%

OHBI: oral health-related behavior index.

The mean number of beneficial behaviors reported by the participant (an OHBI value) was 4.2 (SD 1.6). The distribution of OHBI was found to be practically not skewed (0.09) and had a kurtosis of -0.50. According to the Kolmogorov-Smirnov test, the difference from a normal distribution was 13%. The discrepancy is primarily attributable to the relatively lower frequencies of middle values (4 and 5) compared to a normal distribution.

Most of self-perceived oral health indicators reported by the survey participants correlate positively with our index. Participants with higher OHBI rated their self-perceived oral health better, reported that dental and oral problems did not cause changes in diet, can eat nuts/apple/cucumber/raw carrot without cutting them into pieces, and based on self-assessment felt that no dental treatment is required (Table 4). Correlations of the index with other subjective assessments of oral health, which are not included in the index but essentially measure the same domain, help to provide greater confidence in the index’s performance.

**Table 4:** Spearman correlations between self-perceived oral health characteristics and oral health related behavior index (OHBI).

Characteristic	Parameter	Value
Self-perceived dental and oral health very good	Correlation Coefficient	0.266**
	Sig. (2-tailed)	<0.001
	N	2,085
No new cavities during last year	Correlation Coefficient	0.047
	Sig. (2-tailed)	0.055
	N	1,635
Dental and oral problems have not caused changes in diet	Correlation Coefficient	0.073**
	Sig. (2-tailed)	<0.001
	N	2,230
Possible to eat nuts/apples/cucumbers/raw carrots without cutting them into pieces	Correlation Coefficient	0.226**
	Sig. (2-tailed)	<0.001
	N	2,239
Absence of pain or discomfort in the last couple of years related to teeth or surrounding tissues	Correlation Coefficient	0.039
	Sig. (2-tailed)	0.068
	N	2,238
Absence of bleeding gums	Correlation Coefficient	-0.018
	Sig. (2-tailed)	0.39
	N	2,238
No teeth extracted	Correlation Coefficient	0.011
	Sig. (2-tailed)	0.591
	N	2,236
Based on self-assessment no dental treatment is required	Correlation Coefficient	0.090**
	Sig. (2-tailed)	<0.001
	N	1,923

** Correlation is significant at the 0.01 level (2-tailed).

* Correlation is significant at the 0.05 level (2-tailed).

**Figure 2:**
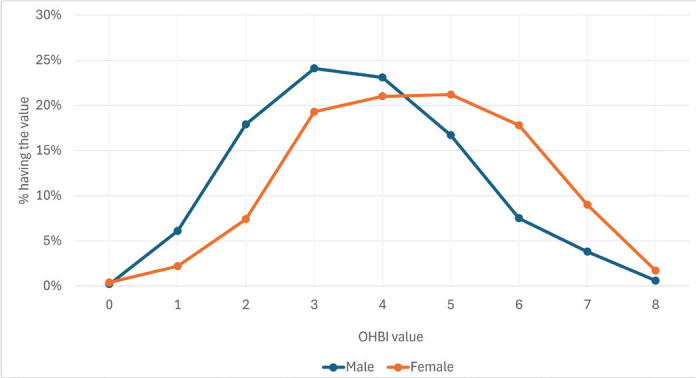
Distribution of the count (from 0 to 8) of beneficial oral health-related behaviors reported by the respondents by gender (%).

Female participants adopted more beneficial oral health-related behaviors than male participants. The difference between the beneficial oral health-related behaviors reported by male and female participants is described in Figure 2. The mean OHBI value in males was 3.67 (SE 0.056; SD 1.55) and 4.52 (SE 0.046; SD 1.59) in females. The difference between means is statistically significant (Student’s t-test, *p* < 0.001).

**Figure 3:**
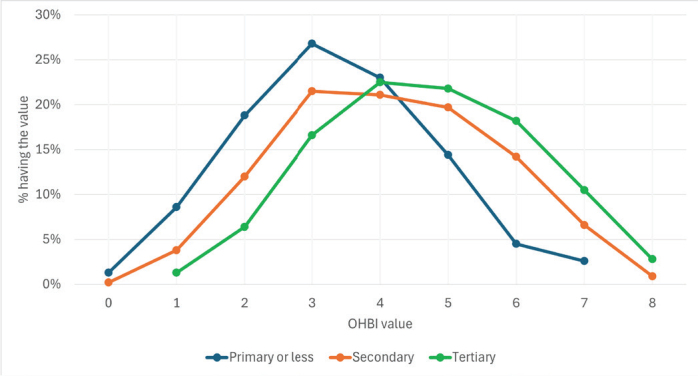
Distribution of the count (from 0 to 8) of beneficial oral health-related behaviors reported by the respondents by educational level (%).

Individuals with higher educational attainment engaged in more beneficial oral health-related activities than those with lower educational levels (Figure 3). The mean OHBI value for educational level primary or less was 3.31 (SE 0.093; SD 1.52); for secondary 4.15 (SE 0.047; SD 1.60) and for tertiary 4.72 (SE 0.065; SD 1.54). The difference between the mean OHBI values at the lower educational levels is statistically significant compared to those at higher levels (Scheffe test, *p* < 0.001 by all pairs).

**Figure 4:**
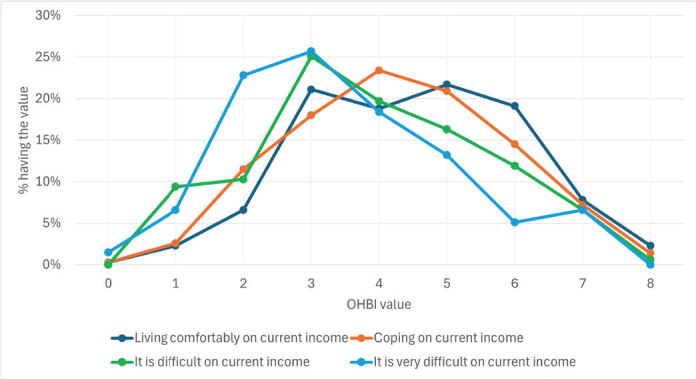
Distribution of the count (from 0 to 8) of beneficial oral health-related behaviors reported by the respondents by household’s income level (subjective assessment ‘Feeling about household’s income nowadays’) (%).

Participants with higher self-estimation of household income level (subjective assessment ‘Feeling about household’s income nowadays’) tended to participate in more advantageous oral health practices than those with lower income levels (Figure 4). The mean OHBI value of the participants who were living comfortably on current income was 4.42 (SE 0.089; SD 1.57); of those who could manage on current income, the mean was 4.29 (SE 0.045; SD 1.60); in the group ‘difficult on current income’ the mean OHBI value was 3.86 (SE 0.097; SD 1.69) and those who find it very difficult on current income the mean OHBI value was 3.50 (SE 0.150; SD 1.67). The difference between mean values in the two higher and two lower household income levels is statistically significant (Scheffe test, *p* < 0.001); however, the differences inside higher and lower income group levels are unsignificant (Scheffe test, corresponding p values are 0.683 and 0.203).

### Socio-economic factors impact on oral health-related behavior

Regression analysis for comparing parameters demonstrates that participants in younger age groups (excluding the 35–44 age range) tend to exhibit more favorable oral health-related behavior compared to the reference category (age 75+). Male participants are more likely to have lower OHBI values than their female counterparts. Higher educational level is associated with higher OHBI values, indicating better oral health-related behavior. Participants residing in the capital city, Tallinn, demonstrate more beneficial oral health-related behavior than those in smaller settlements. Individuals with higher household income levels tend to have higher OHBI values compared to those with lower income levels (Table 5).

**Table 5:** Regression model: socio-economic and demographic characteristics predicting beneficial oral health-related behavior (*n* = 1,992).

Characteristic and Category/Parameter	B	Std. Error	95% Wald Confidence Interval		Hypothesis Test		
			Lower	Upper	Wald Chi-Square	df	Sig.
(Intercept)	3.71	0.2	3.32	4.1	344.2	1	<0.001**
Gender = male	-0.8	0.07	-0.94	-0.67	129.98	1	<0.001**
Gender = female^(a)^		.	.	.	.	.	.
Age group = 35–44	0.05	0.12	-0.19	0.29	0.17	1	0.68
Age group = 45–54	0.33	0.12	0.1	0.56	7.65	1	0.006**
Age group = 55–64	0.24	0.12	0.01	0.48	4.08	1	0.043*
Age group = 65–74	0.41	0.12	0.17	0.66	11.1	1	<0.001**
Age group = 75+ ^(a)^		.	.	.	.	.	.
Language of instruction = Estonian	0.31	0.1	0.11	0.5	9.46	1	0.002**
Language of instruction = Russian^(a)^		.	.	.	.	.	.
Educational level = primary or less	-1.13	0.12	-1.36	-0.9	90.71	1	<0.001**
Educational level = secondary	-0.49	0.08	-0.65	-0.34	37.47	1	<0.001**
Educational level = tertiary^(a)^		.	.	.	.	.	.
Settlement type = capital city (Tallinn)	0.35	0.12	0.12	0.58	8.88	1	0.003**
Settlement type = larger cities (Tartu, Pärnu, Kohtla-Järve, Narva)	0.13	0.11	-0.09	0.35	1.35	1	0.25
Settlement type = other cities, towns	0.13	0.08	-0.03	0.29	2.57	1	0.11
Settlement type = small settlements (villages), countryside^(a)^		.	.	.	.	.	.
Household’s income level (subjective assessment) = living comfortably on current income	0.74	0.16	0.43	1.06	21.17	1	<0.001**
Household’s income level (subjective assessment) = coping on current income	0.75	0.14	0.47	1.03	27.94	1	<0.001**
Household’s income level (subjective assessment) = difficult on present income	0.37	0.16	0.05	0.68	5.22	1	0.02*
Household’s income level (subjective assessment) = very difficult on present income^(a)^		.	.	.	.	.	.

Dependent Variable: OHBI (oral health-related behavior index)

Model: (Intercept); Gender; Age group; Language of instruction;

Educational level; Settlement type;

Household’s income level (subjective assessment

‘Feeling about household’s income nowadays’)

(a) Reference category

Omnibus Test^(b)^

Likelihood Ratio Chi-Square 326.898, df 1, Sig. <.001**

(b) Compares the fitted model against the intercept-only model.

**Table:** Tests of Model Effects

Source	Type III		
	Wald Chi-Square	df	Sig.
(Intercept)	4211.127	1	<0.001**
Gender	129.981	1	<0.001**
Age group	19.451	4	<0.001**
Language of instruction	9.457	1	0.002**
Educational level	93.302	2	<0.001**
Settlement type	9.151	3	0.027*
Income level (subjective assessment)	39.772	4	<0.001**

## Discussion

According to our study, the adherence to beneficial dental health-related behavior in Estonian adults is primarily influenced by gender, educational level, type of settlement, and household income level. Women’s adoption of improved oral health-related behaviors may be linked to their more frequent visits to the dentist. Substantial part of Estonia’s population lives in the capital city, Tallinn, where most dental practices are located; access to dental services in the countryside is limited. In Estonia, adult dental care predominantly operates on a payment basis. The dental care benefit for adults is applied annually at 60 or 105 euros, necessitating personal contributions of 50 and 12.5%, respectively. Fully funded comprehensive public dental care is available to insured individuals with specific systemic health conditions and in instances of emergency dental care. Adult patients are required to bear the cost of their dental care outside these parameters. Given the pricing structure of dental services in Estonia, the household’s income level significantly influences the affordability of such services.

Prior studies have indicated that individuals with lower educational levels and male gender tend to exhibit reduced adoption of preventive oral health behaviors [14, 15]. The same characteristics are often associated with limited use of oral healthcare services [5]. Previous research has illustrated disparities in oral health; the focus should now be pinpointing the elements that contribute to and perpetuate these inequalities and understanding their impact on policy formulation and service provision [18].

The authors propose the inclusion of evidence-based oral health-related education to the national curriculum for basic school and higher educational curriculum, including medicine studies, to raise awareness of oral diseases and related preventive measures. Dentists should recognize their role in not only managing oral pathologies but also in fostering patient education and promoting the adoption of behaviors conducive to oral health to prevent prevalent oral diseases and collaborate with other healthcare providers [19–22]. Consistent oral health education for adults is essential, as poor oral hygiene habits can be transmitted to children, perpetuating the cycle of oral diseases [23, 24].

Our findings highlight the need for targeted age-based preventive measures in Estonia, which focus on improving oral health-related behavior of males and adults aged 35–44 and 75+. Additional services are needed for residents with lower education and income.

The study participants provide a good representation of the adult population in Estonia and can serve as a baseline description of oral health-related practices for further research. The data were gathered using questionnaires, which always carry the risk of respondents leaning towards providing positive and socially acceptable responses [25]. Another limitation related to the survey results is the data gaps (missing values) in the questionnaire responses. We experimented with different imputation methods to fill the data gaps, but as there were no significant differences in the results, we decided to stick to the original data. It is important to note that the data gaps may be also related to more problematic oral health behaviors that respondents may not wish to report. It is also essential to be aware that self-reported oral health-related behavior does not evaluate the quality of oral hygiene, and oral hygiene frequency is not synonymous with oral hygiene quality.

To conclude, to improve the current state of oral health and related behaviors on a national scale, it is essential to incorporate dental services into universal health coverage [1]. The national health insurance fund should redefine specific population-based inclusion criteria for public oral health services that are provided and funded. Additionally, there is a need for consistent oral health education for adults.

## Data Availability

The original survey data stored at the University of Tartu is not publicly available due to agreements made during the research process to protect personal data. Please contact the research team for specific requests or information about the data.
